# Strengthening Borehole Configuration from the Retaining Roadway for Greenhouse Gas Reduction: A Case Study

**DOI:** 10.1371/journal.pone.0115874

**Published:** 2015-01-29

**Authors:** Fei Xue, Nong Zhang, Xiaowei Feng, Xigui Zheng, Jiaguang Kan

**Affiliations:** 1 School of Mines, Key Laboratory of Deep Coal Resource Mining, Ministry of Education of China, China University of Mining and Technology, Xuzhou, Jiangsu 221116, China; 2 Hunan Key Laboratory of Safe Mining Techniques of Coal Mines, Hunan University of Science and Technology, Xiangtan 411201, China; China University of Mining and Technology, CHINA

## Abstract

A monitoring trial was carried out to investigate the effect of boreholes configuration on the stability and gas production rate. These boreholes were drilled from the retaining roadway at longwall mining panel 1111(1) of the Zhuji Coalmine, in China. A borehole camera exploration device and multiple gas parameter measuring device were adopted to monitor the stability and gas production rate. Research results show that boreholes 1~8 with low intensity and thin casing thickness were broken at the depth of 5~10 m along the casing and with a distance of 2~14 m behind the coal face, while boreholes 9~11 with a special thick-walled high-strength oil casing did not fracture during the whole extraction period. The gas extraction volume is closely related to the boreholes stability. After the stability of boreholes 9~11 being improved, the average gas flow rate increased dramatically 16-fold from 0.13 to 2.21 m^3^/min, and the maximum gas flow rate reached 4.9 m^3^/min. Strengthening boreholes configuration is demonstrated to be a good option to improve gas extraction effect. These findings can make a significant contribution to the reduction of greenhouse gas emissions from the coal mining industry.

## Introduction

Many Chinese coal mines are highly gassy with a huge amount of coal mine methane (CMM) occurred in complex geological conditions. CMM is often emitted as a greenhouse gas into the atmosphere during mining operations. CMM has been considered a major mine hazard since mid-19^th^ century when the first documented coal mine gas explosions occurred in the United States in 1810 and in France in 1845 [1]. However, CMM also can be used as a kind of high quality clean energy if this gas is captured and utilized properly. There are three main advantages for recovering CMM in gassy coal seams: improving mine safety, increasing mine economics, and reducing greenhouse gas emissions [2–5].

Gas drainage in Chinese coal mines can be divided into three ways: drainage before mining, drainage during mining, and drainage in the gob. Various drainage methods have been adopted in coal mines, such as laying steel pipes in the waste, creating surface ventholes, and drilling specialized dense cross-measure short holes into roadways as well as the mining panels [6]. However, traditional drainage methods exist some disadvantages, including low gas concentration, high drainage cost, and low utilization rate. Furthermore, gas drainage in complex geological conditions remains difficulty world widely. For example, it is reported that gas extraction rate in the Huainan coal seams is lower because of high in situ gas pressure (greater than 6.2 MPa), high gas content (12~26 m^3^/t), soft coal mass (rigidity coefficient 0.2~0.8), and low permeability (0.001 mD) [7]. Yuan [8] proposed a new method for coal and gas extraction to solve inefficient mining activities in deep, multiseam formations with complex geological conditions. Besides, the dynamic evolution of roof fractures within the mining zones and the governing laws for the vertical fracture-abundant area along the gob side were investigated. In a Y-type ventilation system condition, where the backfill roadway is used as a return airway, Yuan also studied the air pressure field in the gob and the pressure-relieved gas flow law. A new method of coal and gas mining from the retaining roadway by borehole drilling behind the face has been developed and has been proved to be successful in the Huainan coal mines.

Owing to the disturbance in the rock strata above and below the panel after mining, boreholes tend to fracture and the gas extraction rate is poor. Many investigations have been conducted to improve gas drainage effect. Whittles et al. [9] presented geomechanical and gas flow modeling research to provide information on the gas sources and gas flow paths into the face line areas and gate roads. In his follow-up studies [10], a computational model was proposed to predict the stability of methane drainage boreholes. Effects of changes in the roof geology and roadway support system have been investigated. The optimum roadway support system and spacing between boreholes were also evaluated. Karacan et al. [11] studied the influence of borehole diameter, slotted casing length, and casing setting depth on optimizing gas production from gob gas ventholes. Huang et al. [12] and Feng et al. [13] explored the optimization of well location to improve the performance of surface wells for coalbed methane in mining areas. Chen et al. [14] analyzed deformation and failure of a surface venthole casing under compression, tension, and shear conditions using numerical simulation.

In addition to studies on surface ventholes, Karacan et al. [15] investigated different horizontal methane drainage borehole patterns, borehole lengths, and degasification times prior to and during panel extraction to evaluate their effectiveness in reducing methane emissions using a “dynamic” three-dimensional reservoir model. Gentzis et al. [16, 17] proposed a method to quickly assess the stability risk for horizontal wells drilled in coal seams and applied it to the analysis of horizontal borehole stability in the Mist Mountain Formation, SE British Columbia, Canada. Wang et al. [18] summarized the applications and limitations of longhole directional drilling technology for improving gas exploitation.

Although many investigations have been done on borehole stability and gas drainage of traditional drainage methods, little work has been done on cross-measure boreholes drilled in the retaining roadway. Usually, boreholes are often cased with thin, low-strength iron pipe and casing with 10 m depth. Gas drainage rate was poor because the casing may fracture after being retreated from the face and the borehole would become plugged with broken rock. Therefore, it is necessary to optimize borehole configuration for improving the stability of the borehole and gas extraction rate.

This paper presents a field trial project on the gas drainage at the longwall (LW) mining panel 1111(1) of the Zhuji Coalmine, China. The aim of the trial was to investigate the influence of boreholes configuration on borehole stability and gas extraction rate. These findings may provide a useful guidance for drilling design, protection, and extraction scheme.

## Trial Arrangements

### Mining and geological conditions

**Fig 1:**
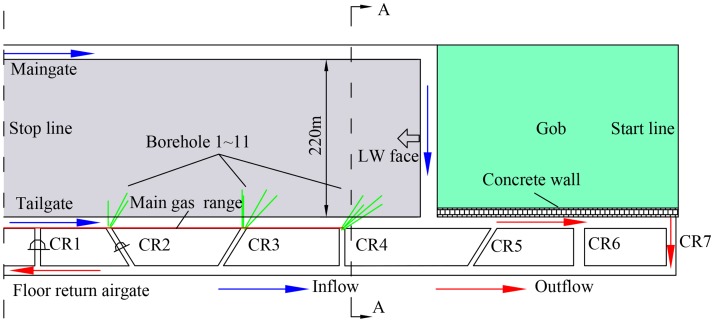
Plan view of panel 1111(1). This figure shows the layout of the face. The boreholes is represented by the green lines. Gray rectangle is coal, and green colored rectangle is gob area.

**Fig 2:**
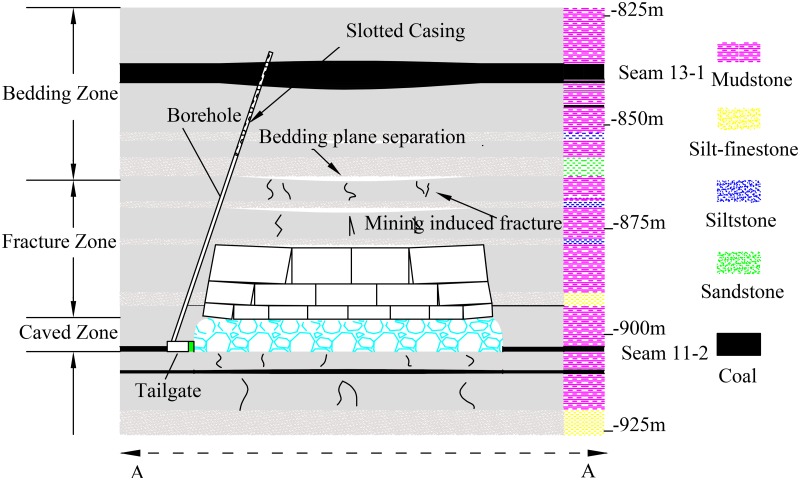
View of A-A section. This figure shows the view of face in the A-A section. Small green rectangle in the tailgate is concrete wall, and blue scope is caved roof.

The trial area is located in panel 1111(1) of the Zhuji Coalmine, operated by the Huainan Mining Group, China. The working panel is in the coal Seam 11–2. It has an average thickness of 1.26 m and an average dip angle of 3°, ranging from 1° to 5°. The mining height for coal is 1.8 m, and the face elevation is -877.6 to -907.0 m, with an average mining depth of 910 m. The panel length is 1612 m and the panel width is 220 m. The working seam is overlain by Seam 13–1, which is outburst coal seam with a thickness of 4 m at a distance of 68 m. As shown in Fig. 1, the face was mined by using retreat LW mining with full seam extraction. It was ventilated by using a Y-type ventilation system with the maingate and inby part of the tailgate being the intake roadways and the outby part of the tailgate and the floor return airgate being the return roadway. There were seven connection roadways (CR) from the start line to the stop line between the tailgate and the floor return airgate. A 3-m-wide concrete wall was constructed behind the LW face to replace the mined-out roadway wall along the gob side for building the retaining roadway. The immediate roof of the panel is mudstone with an average thickness of 9.9 m and the basic roof is fine sandstone and siltstone with an average thickness of 3.2 m. The plan view of this face is shown in Fig. 1. A histogram of the roof and floor lithology is shown in Fig. 2.

### Borehole parameters

**Open-hole parameters**. As shown in Fig. 1, we drilled a total of 11 inclined cross-measure drainage boreholes in three groups sequentially in CR 4, CR 3, and CR 2. The distances separating CR 4, CR 3, and CR 2 from the start line were 840, 1180, and 1460 m, respectively. Each borehole was constructed ahead of the face and drilled from the roof of the CR to the roof of Seam 13–1 (see Fig. 2). Details of the open-hole parameters are listed in Table 1.

**Table 1:** Open-hole parameters of the boreholes.

Number	Diameter (mm)	Azimuth (deg)	Inclination (deg)	Length (m)
1	133	32	58	87
2	133	44	55	93
3	133	49	66	85
4	133	57	62	82
5	133（193）*	57	60	75
6	133（193）	63	56	81
7	133（193）	90	64	76
8	133（193）	90	58	78
9	250	57	56	82
10	250	62	51	83
11	250	90	61	80

The azimuth of the borehole is the angle between drilling direction and axial direction of tailgate. The inclination of the borehole is the angle between drilling direction and horizontal plane.

* Numbers in parentheses represent the diameters of hole sections calculated by adopting dual combined casing.

During the trial, we adopted a ZDY4000S full hydraulic drilling rig produced by the Xian Research Institute of China Coal Technology & Engineering Group, Corp. The rig types offered low speed and large torque, with a maximum torque of 400 N•M and a maximum drilling depth of 350 m.

**Fig 3:**
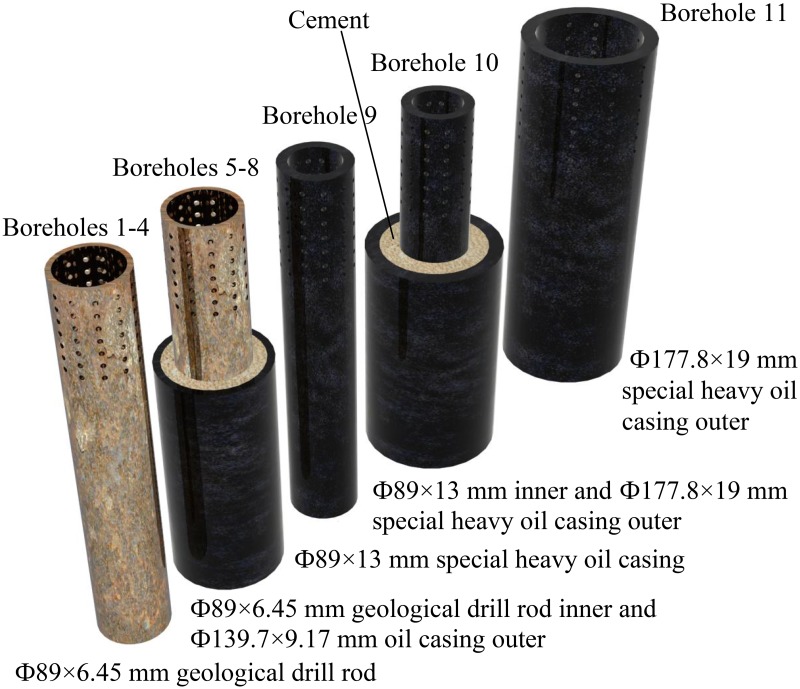
Sketch of borehole configuration.

**Completion parameter**. To ensure the stability and reliability of the borehole, we adopted various casings. As shown in Fig. 3, we used a Ф89×6.45 mm geological drill rod as the casing in boreholes 1~4, dual combined casing (Ф89×6.45 mm geological drill rod inner and Ф139.7×9.17 mm oil casing outer) in boreholes 5~8, and a special heavy oil casing in boreholes 9~11 (Ф89×13 mm and Ф177.8×19mm oil casing). The casing setting depths and slotted casing lengths are listed in Table 2.

**Table 2:** Casing setting depths and slotted casing lengths.

Borehole number	Casing setting depth (m)	Slotted casing length (m)
1	84	15
2	93	27
3	84	25
4	80	10
5	72（15）*	6
6	80（30）	33
7	76（15）	18
8	76（15）	6
9	75	15
10	68（55）	18（18）
11	60	19

* Numbers in parentheses represent casing setting depth and slotted casing length of the casing outer.

After running casing, we inserted an iron pipe of 13 mm in diameter and 4 m in length into the annular space between the casing and the borehole wall as a grouting pipe. Then polyurethane was used to seal the annular space. The sealing depth is not less than 1.5 m. Finally, oil well cementing was used to cement the casing with the borehole wall through the grouting pipe.

### Monitoring plan

**Fig 4:**
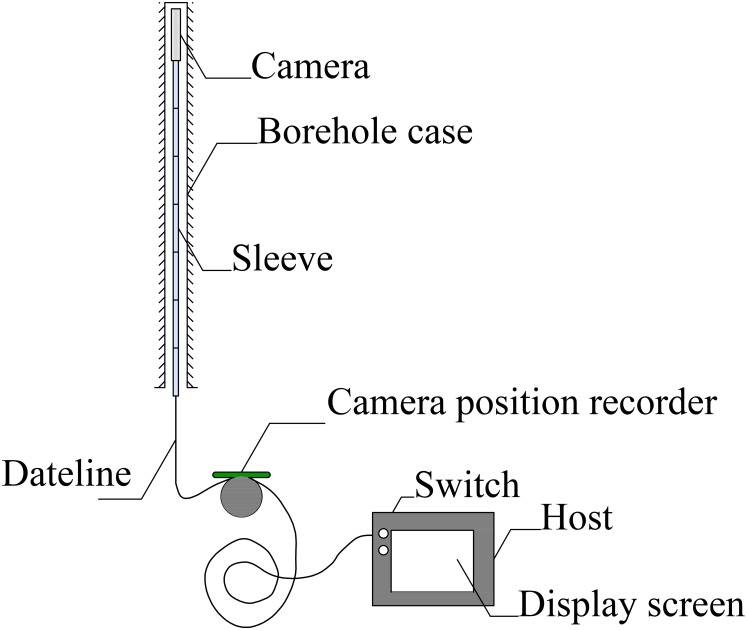
Structure of the BCED.

**Borehole stability**. An instrument called a borehole camera exploration device (BCED) was used to measure borehole stability during the advance of the face. As shown in Fig. 4, it consists of the following basic components [19]: a probe, a data line, a recorder, and a host.

The probe is an angle view camera of 25 mm in diameter and 100 mm in length. It is used to scan the macrofractures or separations at various depths along the borehole.

The data line is a cable with a diameter of 5 mm. It is used to transmit the images or videos from the probe to the host. To insert a probe at any depth into a borehole, we used 100 steel sleeves of 8 mm in inner diameter and 1 m in length. The sleeves are connected by a screw thread.

The recorder is an electronic device. It is used to record the probing depth in the borehole by measuring the cable sliding length.

The host is a control computer. It not only receives and displays the images or videos from the probe but also supplies power from a 3-V battery.

The reading accuracy of the BCED is 0.1 mm, which satisfies engineering requirements. The effective probing depth is 0~100 m. The memory capacity of the host is 4 GB. The BCED can run 8 h on a single charge.

The BCED was used in the following manner. Firstly, the host was opened. Secondly, the probe connected with a sleeve-covered cable was inserted into the borehole slowly. Meanwhile, the scanned videos or images along the borehole and the depth of the probe were transmitted to and stored in the host. Thirdly, deformation or fracture of the casing could be viewed on a display screen in real time and the information stored could be transmitted from the host to a processing computer.

When the distance between the face and the borehole was 30 m, stability monitoring began and performed once a day.

**Gas extraction effect**. The ZD41200 gas pipeline with a multiple parameter measurement device (GPMPMD) was used to monitor the gas concentration and flow rate of boreholes 1~11.

The ZD41200 GPMPMD can directly display multiple gas parameters in the pipeline under normal conditions. Following parameters are included in this apparatus: volume flow rate, pressure, temperature, methane concentration, and carbon monoxide concentration. The ZD41200 GPMPMD is a high-tech detection device capable of displaying various parameters in real time and includes a methane and carbon monoxide concentration overrun acousto-optic alarm. This device can be used alone in drainage pipelines on the ground or underground. It can also output a standard signal of 200~1000 Hz to connect conveniently to a variety of coal mine safety production monitoring systems.

**Fig 5:**
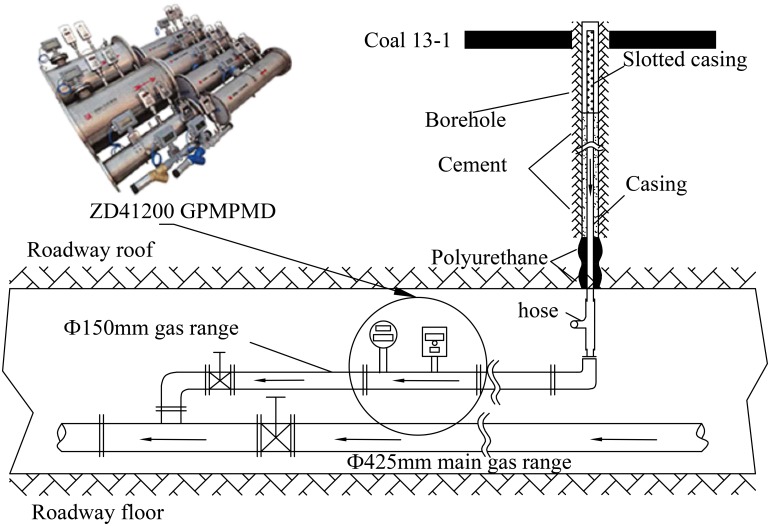
Scheme for gas drainage effect monitoring.

As shown in Fig. 5, a hose was used to connect the borehole to one gas range of 150 mm in diameter. The range has one ZD41200 GPM-PMD before leading into the main gas range of the panel. Monitoring data can be transmitted through a cable to the ground machine for viewing and analyzing.

## Results and Discussion

### Borehole stability

**Fig 6:**
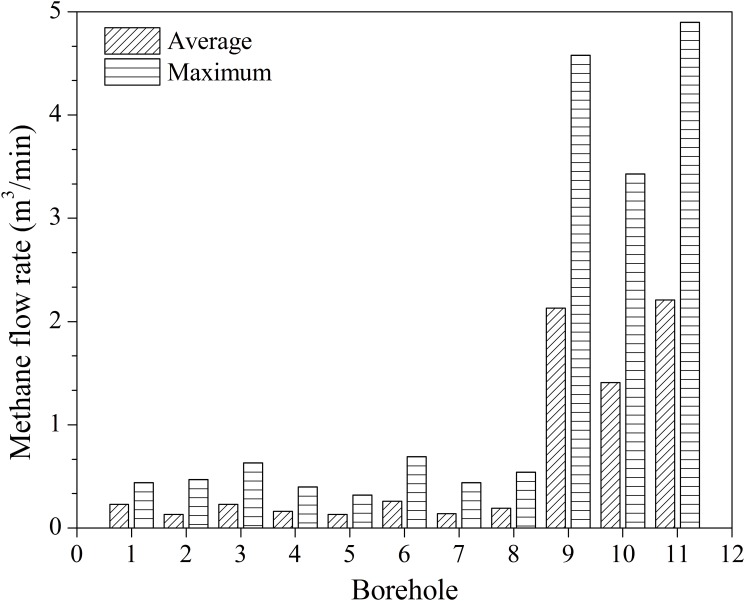
Data for borehole failure position along the casing, height into the roof, and distance behind the coal face where casing has failed. Legends: dense slash is distance behind the coal face; horizontal is distance along the casing; sparse slash is height into roof.

**Fig 7:**
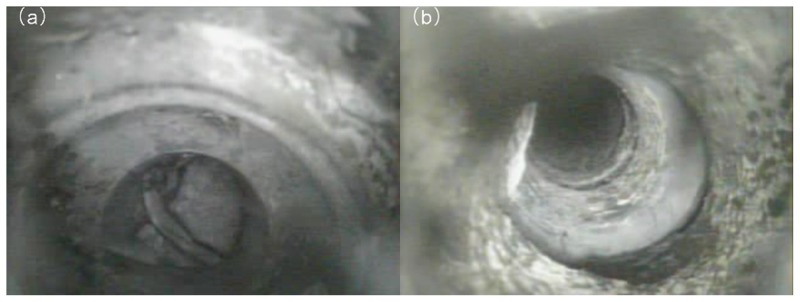
Photographs of borehole 6 and 8 failure sections. The Figure have two parts: a) Borehole 6 failure section; b) Borehole 8 failure section.

After five months of monitoring, boreholes 1~8 were found to be broken, whereas boreholes 9~11 did not fracture during the whole extraction. Details of the borehole failure position along the casing, the height into the roof, and the distance behind the coal face where the casing failed are shown in Fig. 6. Borehole 6 and 8 failure sections are shown in Fig. 7.

From Fig. 6 we can conclude the following:

1. Boreholes 1~8 were broken at a distance of 2~14 m behind the coal face, and 87.5% of that occurred over a distance of 9~14 m behind the coal face. This is consistent with the observed value of the coal face periodic weighting pace, which is about 15m. It implies that the stress disturbance from the periodic breakage of the basic roof is the main reason for borehole rupture.

2. The failure position of boreholes occurred at depths of 5~10 m along the casing and 62.5% of failures happened at the casing connection. This suggests that the casing joint area easily undergo fracture, because of lower strength than the casing body, and thus it is necessary to strengthen the casing joint zone. For example, premium casing connectors can be adopted to strengthen the casing joint. [20]

3. The failure position of the boreholes all occurred at heights of 4~9 m from the roof surface. From the comprehensive geological histogram panel in Fig. 2, we can see that borehole failure all occurred in the mudstone immediate roof. Because mudstone is a weaker material than sandstone and siltstone, the stress concentration around the boreholes in mudstone may make them to fail easily. In addition, the stresses created by mining activities in the coal can lead to additional stresses and displacements in the formations that surround the boreholes, which can lead to borehole failure [3].

Comparing the monitoring results of boreholes 1~8 with those of boreholes 9~11, it can be concluded that the optimization of boreholes configuration can effectively improve the stability of the borehole. Adopting a special thick-walled casing is more effective on improving borehole stability than adopting a dual combined casing.

### Gas extraction effect

**Fig 8:**
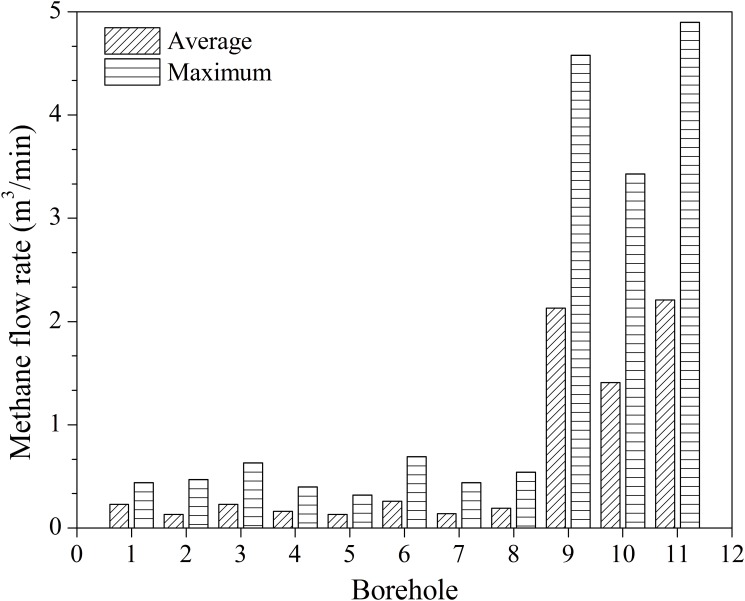
Average and maximum methane flow rates of boreholes 1~11. Legends: slash is average methane flow rate; horizontal is maximum methane flow rate.

The average and maximum methane flow rates for boreholes 1~11 are contrasted in Fig. 8. Considering the borehole stability monitoring results, it is concluded from Fig. 8 that gas extraction rate is closely related to borehole stability. As borehole stability is improved for boreholes 9~11, the average methane flow rate increased 16-fold from 0.13 to 2.21 m^3^/min, and the maximum methane flow rate increased 14-fold from 0.32 to 4.9 m^3^/min.

Therefore, optimizing the borehole configuration to enhance the stability of the borehole can improve the efficiency of gas extraction, reduce the number of holes that need to be drilled, and save construction costs.

In addition, by comparing boreholes 9 and 11, it can be seen that, once borehole stability is guaranteed, increasing the borehole diameter has little influence on improving gas extraction. The diameter of borehole 11 relative to that of borehole 9 has increased 1.2-fold, while the average and maximum methane flow rates only increased by 4% and 7%, respectively.

**Fig 9:**
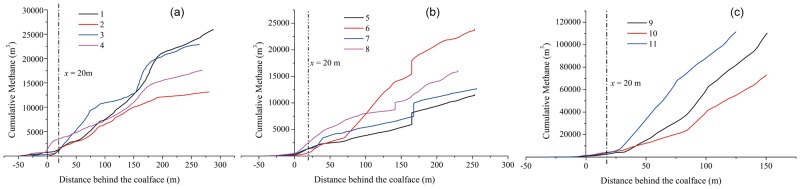
Plots of cumulative methane flow of boreholes 1~11 with face advancing. The negative value of distance represent boreholes is ahead of the working face. The Figure have three parts: a Boreholes 1~4; b Boreholes 5~8; c Boreholes 9~11.

Plots of the cumulative methane flow rate of boreholes 1~11 with distance from the coal face are shown in Fig. 9. It can be seen that, because of the stress relief in the overlying coal seam from mining, the curves of boreholes 9~11 begin to rise rapidly at a distance of 20 m behind the coal face and maintain rapid growth until the panel finished. The average cumulative methane flow of boreholes 9~11 reaches 98265 m^3^ after only 35 days of drainage. In contrast, gas extraction efficiency of boreholes 1~8 is low and the curve shows slow growth due to casing fracture and the borehole being plugged by broken rock. The average cumulative methane flow of boreholes 1~8 is only 17984 m^3^. It can also be concluded that the lagging distance of stress relief in the overlying coal seam is 20 m under similar engineering geological conditions.

## Conclusions and Future work

A field trial of strengthening borehole drilling configuration from the retaining roadway for improving extraction rate was conducted at the Zhuji Coalmine. In the trail the evolution of borehole destruction over time and space was measured through borehole stability monitoring method. Borehole failure mainly occurred at a distance of 9~14 m behind the coal face, which is in accordance with the observation value of the coal face periodic weighting pace. It implies that the stress disturbance from the periodic breakage of the basic roof is the main cause of borehole rupture. The failure position of boreholes occurred in the mudstone roof at heights of 4~9 m, indicate that the stress concentration around the boreholes in soft rocks is higher than that in hard rocks. In addition, the observations also show that the casing joint area more easily experiences failure than the casing body, which makes it necessary to strengthen the casing joint. Adopting a special thick-walled casing is more effective for improving borehole stability than adopting a dual combined casing.

Gas flow monitoring results show that gas extraction rate has a close relationship with borehole stability. When borehole stability is improved, the average methane flow rate increased 16-fold and the maximum methane flow rate increased 14-fold. Increasing the borehole diameter has little influence on improving gas extraction rate. It can also be concluded that the lagging distance of stress relief in the overlying coal seam is 20 m under similar engineering geological conditions.

These results demonstrate that optimizing borehole configuration can effectively enhance the stability of the drilling, thereby significantly improving gas extraction. The findings can provide a useful guidance for drilling design and protection and has important theoretical and practical significance for reducing greenhouse gas emissions as well as preventing gas disaster.

However, due to the inhomogeneity of rock and coal and the complexity of engineering geological conditions, the stresses on the borehole casing changes along the casing axis. Consequently, the next step should be conducted further research to analyze the stress distribution within the borehole casing along the axis combined with theoretical analysis and numerical simulation. Furthermore, the slotted casing length and casing setting depth have a great influence on optimizing gas production and should be taken into account.
